# Fronto-limbic disconnection correlates with paroxysmal sympathetic hyperactivity following traumatic brain injury: An indirect disconnection-symptom mapping study

**DOI:** 10.1016/j.nicl.2025.103937

**Published:** 2025-12-23

**Authors:** Eric W Moffet, Sancharee Hom Chowdhury, Ediel Almeida, Xiangxiang Kong, Lujie Chen, Jiachen Zhuo, Nicholas A Morris, Gunjan Y Parikh, Neeraj Badjatia, Jamie E Podell

**Affiliations:** a University of Maryland School of Medicine, 655 W Baltimore St, Baltimore, MD 21201, USA; b University of Maryland, Baltimore County, 1000 Hilltop Cir, Baltimore, MD 21250, USA

**Keywords:** Traumatic brain injury, Paroxysmal sympathetic hyperactivity, Susceptibility weighted imaging, Indirect disconnection-symptom mapping

## Abstract

Paroxysmal sympathetic hyperactivity (PSH) is a clinically important manifestation of dysautonomia following traumatic brain injury (TBI). While it is thought to arise from central autonomic network disconnection, supporting evidence is limited. Here, we integrate clinically obtained magnetic resonance imaging (MRI) lesion data with human connectome data to identify specific white matter tract disconnections and gray matter parcel damage associated with PSH. Our sample included 117 patients who underwent susceptibility weighted imaging and 3D T1 MRI sequences as part of clinical care while admitted at our institution between January 1, 2016 and July 1, 2018. Susceptibility lesion masks were manually created and registered to standard template space. High quality registrations were obtained in 96 patients (50% with PSH), who were included in the study. Using the Matlab Lesion Quantification Toolkit, we assessed white matter tract disconnection severity and gray matter parcel damage for each patient. We compared results according to a binary PSH clinical diagnosis using Wilcoxon rank sum tests and a standard ordinal PSH diagnostic likelihood score (with 0–11 range) using Pearson correlations, Bonferroni-corrected for multiple comparisons. PSH diagnosis was associated with greater disconnection severity in nine tracts, two of which also correlated with higher diagnosis likelihood: the right uncinate fasciculus and the anterior corpus callosum. Damaged parcels associated with PSH included left prefrontal regions of the default mode network and the ventral salience network. In summary, our work implicates disconnection of fronto-limbic components of the central autonomic network in the pathophysiology of TBI-related PSH.

## Highlights

• Paroxysmal sympathetic hyperactivity (PSH) often complicates traumatic brain injury recovery.
• PSH is associated with diffuse axonal injury, identifiable on susceptibility-weighted MRI.
• We integrated susceptibility-weighted lesions with human connectome data and PSH measurements.
• Right uncinate fasciculus and anterior corpus callosum disconnections correlated with PSH.

## 1 Introduction

Paroxysmal sympathetic hyperactivity (PSH) is a clinically and prognostically important manifestation of dysautonomia that frequently occurs after traumatic brain injury (TBI). Affected patients experience longer intensive care unit (ICU) stays, increased infectious, cardiac and renal complications, higher mortality, and worse functional outcomes (Fernandez-Ortega et al., 2012, Hendricks et al., 2010, Khalid et al., 2019, Mathew et al., 2016). PSH is characterized by recurrent episodes of sweating, posturing, tachycardia, tachypnea, hypertension and hyperthermia (Baguley et al., 2014). The most widely accepted pathophysiologic framework – the excitatory-inhibitory ratio model – proposes that disconnection within the central autonomic network impairs top-down control over brainstem and spinal autonomic centers, rendering them hyperexcitable to afferent stimuli (Baguley, 2008).

Neuroimaging evidence supporting this model has been limited by inconsistent terminology, small sample sizes, and challenges performing research-quality MRI scans in critically ill TBI patients. We previously identified initial computed tomography (CT) features independently predictive of PSH, including the presence of subarachnoid or intraventricular hemorrhage, cisternal compression, and diffuse axonal injury (Podell et al., 2021). MRI offers greater sensitivity to detect diffuse axonal injury, the strongest CT predictor of PSH (Podell et al., 2021), particularly with hemorrhage-sensitive sequences such as susceptibility weighted imaging (SWI). In a subsequent qualitative MRI study, we observed that SWI lesions within pre-defined central autonomic network regions – including the medial temporal lobes, anterior cingulate/medial prefrontal cortex, and the corpus callosum – are associated with increased PSH likelihood (Podell et al., 2024). However, this analysis relied on *a priori* hypotheses derived from studies of autonomic function in healthy individuals and patients with epilepsy or stroke (Wang et al., 2024, Seifert et al., 2015, Kim et al., 2023, Reisert, 2021) and did not allow for data-driven discovery of PSH-specific injury patterns.

Given the excitatory-inhibitory ratio hypothesis, advanced imaging techniques such as diffusion tensor imaging tractography and functional MRI (fMRI), which directly assess neural network structural and functional connectivity, might elucidate a PSH network. However, techniques are rarely included in standard clinical MRI protocols and substantially increase scan time, limiting safety and feasibility in acutely ill TBI patients. This is particularly relevant for patients at risk for PSH, who may have multiple medical comorbidities requiring active ICU care. However, standard clinical MRI sequences that assess for CT-occult ischemia or microhemorrhages are often performed acutely (Podell et al., 2024). Indirect disconnection-symptom mapping offers an innovative approach that leverages these routine sequences to approximate advanced connectivity analyses (Griffis et al., 2021, Sperber et al., 2022). Using this method, visible MRI lesions are integrated with population-based human connectome data to predict structural and functional neural network disconnections based on lesion location in a standard imaging space. Clinical or behavioral symptom data can then be used as explanatory variables to identify specific white matter tract disconnections or functionally-defined gray matter parcel damage associated with the symptom of interest (Griffis et al., 2021, Irimia et al., 2012, Sperber et al., 2022). Importantly, indirect analyses have demonstrated similar or even improved correlations with behaviorally relevant symptoms compared to direct advanced neuroimaging analyses (Tozlu et al., 2021).

In this study, we applied indirect disconnection-symptom mapping using an open source Matlab toolkit to identify white matter tract disconnection severity and gray matter parcel damage associated with PSH in critically ill TBI patients who underwent an MRI of the brain as part of clinical care. We hypothesized that this technique would facilitate the discovery of specific white matter tract disconnections implicated in PSH, in keeping with the excitatory-inhibitory ratio model.

## 2 Methods

### 2.1 Study design

We performed a retrospective case-control study investigating the neuroanatomic correlates of PSH in an enriched cohort of hospitalized patients with TBI requiring ICU admission. Included patients must have undergone an MRI of the brain as part of clinical care and had a hospital stay of at least two weeks. The study was approved by the Institutional Review Board of the University of Maryland, Baltimore, which waived the need for informed consent.

### 2.2 Patient identification

**Fig. 1:**
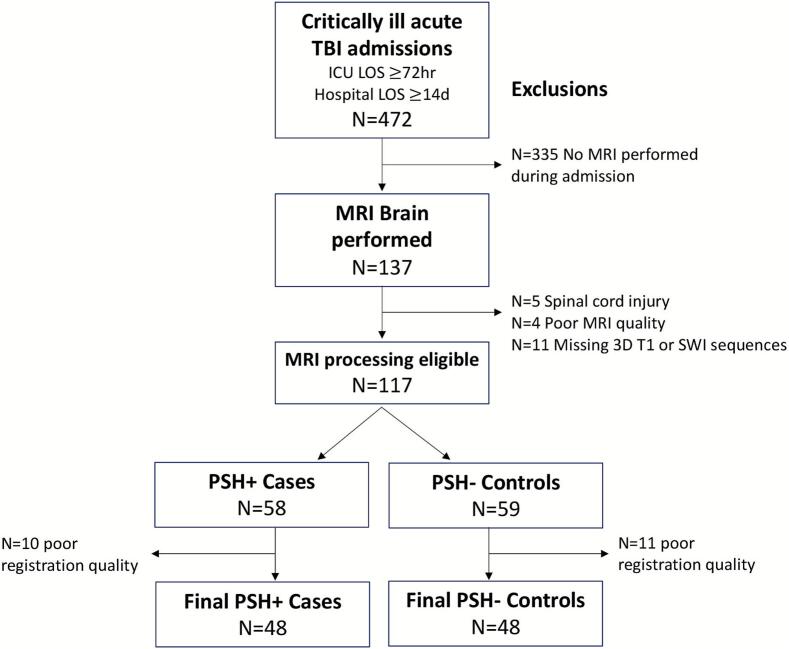
**Patient Flow Diagram.** Patients were identified via trauma registry query for direct admissions for traumatic brain injury (TBI) with intensive care unit (ICU) length of stay (LOS) of at least 72 h and hospital LOS of at least 14 days. We included only patients for whom a magnetic resonance imaging (MRI) of the brain was performed as part of clinical care and included useable 3D T1 and susceptibility weighted imaging (SWI) sequences. Diagnosis of paroxysmal sympathetic hyperactivity (PSH) was determined based on standard first-line medication administration corroborated by documentation of the indication and PSH-assessment measure (PSH-AM) scores.

We included critically ill TBI patients who were admitted to the ICU at a Level 1 Trauma Center/Primary Adult Resuscitation Center from January 1, 2016 to July 1, 2018 (Fig. 1). Patients were identified using an institutional trauma registry, screening for: Head Acute Injury Scale (AIS) > 0, ICU stay ≥ 72 h, hospital stay ≥ 14 days, and MRI Brain performed during hospitalization, which included SWI and 3D T1 sequences. The SWI sequence was our primary focus in this study based on our prior work suggesting a strong link between diffuse axonal injury and hemorrhagic lesions and PSH (Podell et al., 2024, 2021). Patients with concomitant spinal cord injury were excluded due to high likelihood of confounding autonomic manifestations. Patients were further excluded for poor-quality MRI data or poor-quality image registrations during first-level processing (Fig. 1).

### 2.3 Clinical/demographic data collection

An institutional trauma registry was utilized to extract injury and demographic information: age, sex, race, mechanism and type of injury, Head Abbreviated Injury Scale (AIS), Injury Severity Score (ISS), Trauma ISS, Glasgow Coma Scale (GCS) upon ICU admission and hospital discharge, intracranial pressure monitor placement (and type of monitor used), operative neurosurgical procedures, total ICU and hospital length of stays (LOS), duration of mechanical ventilation, and mortality. Electronic Health Records (EHRs) were then reviewed to determine PSH diagnostic features (details below) and the hospital day on which the MRI brain was performed.

### 2.4 PSH characterization

As in our prior work, the binary clinical diagnosis of PSH was determined based on the administration of institutional first-line treatments for PSH (propranolol and/or bromocriptine) for at least 3 consecutive days, with indication confirmed by daily progress notes with terminology such as PSH, “storming,” “autonomic dysfunction,” “diencephalic seizures/storms,” “paroxysmal autonomic instability with dystonia, PAID,” etc., as in prior work (Chowdhury et al., 2025, Podell et al., 2024, Podell et al., 2021, Salasky et al., 2024). Detailed chart review confirmed that both propranolol and bromocriptine were used in this patient population exclusively for brain injury-related dysautonomia. This case-control definition is inclusive and pragmatic, identifying patients with symptoms clinically significant enough to warrant pharmacotherapy.

Our second PSH characterization method involved retrospective PSH-Assessment Measure (PSH-AM) Diagnostic Likelihood Tool (DLT) scoring (Table 1). Based on our previous work demonstrating the strongest imaging correlations with PSH-AM DLT scores (Podell et al., 2024), we focus on the DLT score as an ordinal measure of PSH diagnosis likelihood. This score is tabulated based on presence or absence of 11 criteria agreed upon by an expert consensus panel to indicate increasing certainty of PSH diagnosis(Baguley et al., 2014).

**Table 1:** Paroxysmal sympathetic hyperactivity-assessment measure (PSH-AM).

**Clinical feature scale (CFS)**					
	0	1	2	3	Score
Heart rate	<100	100–119	120–139	≥ 140	
Respiratory rate	<18	18–23	24–29	≥ 30	
Systolic blood pressure	<140	140–159	160–179	≥ 180	
Temperature	<37	37–37.9	38–38.9	≥ 39	
Sweating	Nil	Mild	Moderate	Severe	
Posturing	Nil	Mild	Moderate	Severe	
			**CFS subtotal**		
**Diagnosis likelihood tool (DLT)**					
Clinical features occur simultaneously					
Episodes are paroxysmal in nature					
Sympathetic over-reactivity to normally non-painful stimuli					
Features persist ≥ 3 consecutive days					
Features persist ≥ 2 weeks					
Features persist despite treatment of alternative differential diagnoses					
Medication administered to decrease sympathetic features					
≥ 2 episodes daily					
Absence of parasympathetic features during episodes					
Absence of other presumed cause of features					
Antecedent acquired brain injury					
			**DLT subtotal**		
**Combined total (CFS + DLT)**			Unlikely	<8	
			Possible	8–16	
			Probable	≥ 17	

### 2.5 Radiographic data collection

All patients underwent an MRI of the brain at the discretion of their clinical team. Scans were completed with parallel imaging technology on either a 3 T Siemens Tim Trio Scanner or a 1.5 T Siemens Avanto (Siemens Medical Solutions; Erlangen, Germany). A standard institutional TBI MRI protocol was utilized for all patients, which included SWI and 3D T1 sequences, used for lesion tracing and registration in this study, respectively. Parameters for SWI were TE = 20 ms, TR = 28 ms, flip angle = 15°, FOV = 320 mm, resolution = 0.8 × 0.7 × 2 mm^3^, and 72 axial slices for the 3 T scanner; TE = 40 ms, TR = 50 ms, flip angle = 20°, FOV = 512 mm, resolution = 0.5 × 0.5 × 2 mm^3^, and 88 axial slices for the 1.5 T scanner. Parameters for 3D T1 were TE = 3.44 ms, TR = 2 s, flip angle = 9°, FOV = 256, resolution = 0.9 × 0.9 × 2 mm^3^, and 72 axial slices for the 3 T scanner. For the 1.5 T scanner TE = 4.76 ms, TR = 11 ms, flip angle = 20°, FOV = 256, resolution = 0.9 × 0.9 × 2 mm^3^, and 88 axial slices.

### 2.6 Summary statistics

Descriptive statistics were completed for demographic, injury and inpatient clinical characteristics. Frequencies were calculated for categorical variables, means and standard deviations for normally distributed continuous variables, and medians with 25th and 75th percentiles for non-normally distributed continuous or ordinal variables. Data was stratified by PSH diagnosis, and group-wise comparisons were made using two-sample t-tests, Wilcoxon rank sum tests, or Fisher’s Exact or Chi Squared tests, where appropriate. A p-value of < 0.05 was considered statistically significant with a Bonferroni correction for multiple comparisons for second-level imaging analyses of white matter tract disconnection severity (70 comparisons; α_adjusted_ = 0.00071) and gray matter parcel damage (135 comparisons; α_adjusted_ = 0.00037).

### 2.7 First-level radiographic data processing and analyses

Clinical images were converted and deidentified from DICOM to NIFTI format using dcm2niix. Raw images were checked for quality and completeness of 3D T1 and SWI sequences for inclusion in the study. Intraparenchymal lesion tracing was performed on SWI sequences using ITK-SNAP (https://www.itksnap.org/pmwiki/pmwiki.php?n = Documentation.HomePage) software. Authors (EWM, EA) hand-painted intraparenchymal SWI lesions on each slice. Additional sequences, including T1, DWI, and FLAIR, were co-registered in ITK-SNAP to assist with lesion identification. The painted layers resulted in a 3D binary mask for each patient, which was reviewed, edited, and finalized by the senior author (JEP) to ensure accuracy and consistency. Artifacts related to intracranial pressure (ICP) monitors and external ventricular drains (EVDs) were excluded from the lesion masks as part of this editing process. Each mask was then registered via linear transformation to that patient’s 3D T1 image using FSL’s FLIRT tool (https://fsl.fmrib.ox.ac.uk/fsl/docs/#/). Each patient’s 3D T1 was registered to MNI template space using both linear and non-linear warping via FLIRT and FNIRT, respectively (https://fsl.fmrib.ox.ac.uk/fsl/docs/#/). The resulting transformation functions were applied to both the SWI (for quality control purposes) and the SWI lesion mask (for input to the lesion quantification toolkit).

Registration quality was then checked manually for each subject by the senior author (JEP) using ITK-SNAP. The MNI template image was opened as the main image, along with a single patient’s MNI-registered T1 image and MNI-registered SWI image. The MNI-registered, binarized lesion mask was added as a segmentation overlay. Image registration quality was coded as acceptable, poor, or failed (Supplementary Fig. 1). Only acceptable images were further analyzed; subjects with poor or failed registrations were excluded from the study.

**Fig. 2:**
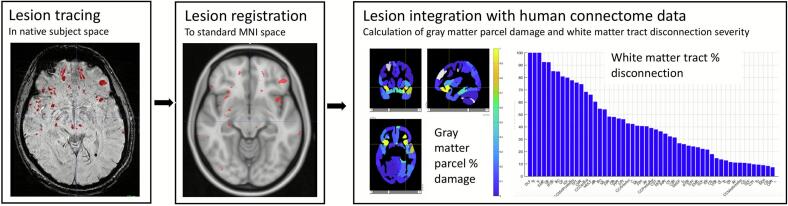
**Lesion processing pipeline for indirect disconnection mapping.** Hemorrhagic lesions identified on susceptibility weighted imaging (SWI) sequences were manually traced in native subject space using ITK-SNAP in order to create a 3D binary lesion mask, which was registered to the patient’s 3D T1 scan and normalized to a standard MNI space. After checking registration quality, normalized lesion maps were integrated with human connectome data using the Lesion Quantification Toolkit to yield information regarding the lesion’s impact on white matter tract disconnections and functionally-defined gray matter parcel damage.

MNI-registered, quality-assured SWI lesion masks were then integrated with population based structural and function connectome data using the Lesion Quantification Toolkit, an open-source program that runs in Matlab (Griffis et al., 2021). This toolkit utilizes publicly available atlases of white matter tracts to estimate disconnection severity and gray matter parcels to estimate percent damage to pre-defined regions induced by focal brain lesions. Outputs of interest for each subject that underwent further second-level analysis included white matter tract disconnection severities (a value of 0 to 100 % for each white matter tract) and gray matter parcel lesion loads (a value of 0 to 100 % for each gray matter parcel). The overall imaging and lesion processing workflow for an individual patient, including a visualization of the lesion quantification toolkit outputs of interest is illustrated in Fig. 2.

For white matter tract disconnection severity measurements, we utilized the toolkit’s default HCP-842 white matter tract atlas (Yeh et al., 2018). This atlas is based on directional diffusion information averaged from 842 neurotypical subjects to generate 500,000 streamline trajectories, grouped neuroanatomically into 65 white matter tracts. While the original HCP-842 atlas considered the corpus callosum as a single tract, the lesion quantification toolkit further divides the corpus callosum into five distinct anterior-posterior regions using the FreeSurfer wmparc atlas (https://surfer.nmr.mgh.harvard.edu/fswiki). Thus, disconnection severity is measured across 70 distinct white matter tracts. The lesion quantification toolkit estimates disconnection severity by determining the number of streamlines from each tract that intersect with the lesion volume and then divides that number by the total number of streamlines comprising each tract, to yield a percent disconnection severity. By this method, streamlines can be considered analogous to axon bundles. Accordingly, a lesion that transects a streamline at one point has the same effect as one that covers the entire length of a streamline.

For gray matter parcellation, we used the default “Schaefer Atlas,” which segments regions by function (Schaefer et al., 2018). This atlas is derived from task-based and resting state fMRI data of 1489 neurotypical subjects, resulting in 135 pre-defined grey matter regions, categorized into one of seven functional networks. In contrast to tract disconnection severity, gray matter parcel damage is a true lesion load, determined based on the number of lesion voxels overlapping with each atlas-defined parcel, divided by the total number of voxels comprising each parcel.

### 2.8 Second-level radiographic data analysis

#### 2.8.1 Quantitative analysis of LQT data

We assessed disconnection severity differences across 70 white matter tracts in PSH cases versus controls using Wilcoxon rank sum tests. Our statistical significance threshold was Bonferroni corrected for multiple comparisons; results from regions meeting this strict criterion are reported, with uncorrected *p*-value results listed. Linear relationships between white matter tract disconnection severity and PSH-AM DLT scores (ordinal value of 0–11; Table 1) were also investigated using Pearson correlations and were reported for all regions with statistically significant Wilcoxon rank sum test results. We similarly assessed and reported gray matter parcel damage results for the 135 tested regions.

#### 2.8.2 LQT data visualization

To display the distribution of hemorrhagic lesions in our sample, all MNI-registered lesion masks were added as overlays onto the same image and visualized using MRICron (Rorden, 2025). White matter tracts with significant differences in disconnection severity and gray matter parcels with significant differences in percent damage between cases and controls were also displayed using DSI Studio (Yeh, 2025) (Version 2022 Hou), separated according to white matter tract type (Association, Commisural, and Projection) for easier visualization.

## 3 Results

### 3.1 Description of included patients with high quality imaging results

Our sample consisted of 117 acute critically ill TBI patients whose brain MRI’s were suitable for initial processing (Fig. 1). This enriched sample included n = 58 (50 %) who were clinically diagnosed with PSH. Registration to MNI template space failed in 3 patients (3 %) and was of poor quality in another 21 (18 %). Exclusions for poor registration were distributed equally across cases (17 %) and controls (19 %). Examples of acceptable, poor, and failed registrations are provided in the Supplementary Material. The final included sample consisted of 96 total patients, 48 (50 %) with PSH and 48 (50 %) without PSH.

Table 2 compares demographic, injury, and clinical data across PSH cases and controls for the final included sample. Significant between-group differences included younger age for PSH patients as well as more motor vehicle collisions (MVC) and fewer falls. Also, the PSH group was less likely to be following commands upon emergency department discharge and had a lower total GCS score at that time. PSH patients were also more likely to have an EVD placed.

**Table 2:** Demographic and clinical data features according to paroxysmal sympathetic hyperactivity diagnosis.

**Characteristics**	**PSH** **N = 48**	**Control** **N = 48**	**P-value**
Age, Year median (25th, 75th %)^a^	25.5 (22, 41.5)	59.5 (44.5, 72.25)	<0.001
Sex, M (%)^b^	41 (85.42)	34 (70.83)	0.08
Race^b^			
− Asian, n (%)	1 (2.1)	1 (2.1)	1
− Black, n (%)	18 (37.5)	19 (39.6)	0.83
− White, n (%)	20 (41.7)	26 (54.2)	0.22
− Other, n (%)	9 (18.8)	2 (4.2)	0.025
Injury Mechanism^b^			
− Assault, n (%)	3 (6.3)	5 (10.4)	0.46
− Fall, n (%)	4 (8.3)	14 (20.9)	0.009
− MCC, n (%)	6 (12.5)	6 (12.5)	1
− MVC, n (%)	27 (56.3)	10 (20.8)	<0.001
− Other/Unknown, n (%)	2 (4.2)	4 (8.3)	0.40
− Pedestrian, n (%)	6 (12.5)	9 (18.8)	0.40
DLT score, median (25th, 75th %)^a^	9 (8, 9.25)	3 (1, 4)	<0.001
Hospital day of MRI, median (25th, 75th %)^a^	4 (3, 9.25)	5 (2, 10.25)	1
Hospital day of PSH diagnosis, median (25th, 75th %)^a^	5.5 (4, 8.25)	n/a	n/a
GCS at ICU admission, median (25th, 75th %)^a^	6 (5, 7)	7 (5.75, 11.5)	0.009
GCS_Motor at ICU admission, median (25th, 75th %)^a^	4 (3, 5)	5 (3.5, 6)	0.087
Following commands at ICU admission, yes (%)^b^	5 (10.42)	17 (35.42)	0.004
Neurosurgical intervention, yes (%)^b^	9 (18.75)	12 (25.00)	0.459
EVD, yes (%)^b^	41 (85.42)	16 (33.33)	<0.001
Duration of MV, days median (25th, 75th %)^a^	13 (10, 16)	12 (7, 19)	0.76
ICU LOS, days median (25th, 75th %)^a^	18.7 (14.3, 24.5)	17.7 (13.3, 23.0)	0.704
Hospital LOS, days median (25th, 75th %)^a^	26.5 (24.3, 38.8)	24.5 (19.3, 33.2)	0.082
In-hospital mortality, yes (%)^b^	2 (4.17)	6 (12.5)	0.14
GCS at hospital discharge, median (25th, 75th %)^a^	11 (10, 14)	14 (11, 15)	0.096
GCS_Motor at hospital discharge, median (25th, 75th %)^a^	6 (5, 6)	5 (3.5, 6)	0.22
Hospital discharge Following Commands, yes (%)^b^	28 (60.87)	34 (77.27)	0.093

Frequencies shown for categorical variables. Medians & percentiles shown for continuous, non-normally distributed variables. Wilcoxon rank sum^a^ and Chi Squared^b^ tests were performed where appropriate. PSH: paroxysmal sympathetic hyperactivity; MCC: motorcycle collision; MVC: motor vehicle collision; DLT: diagnosis likelihood tool; MRI: magnetic resonance imaging; ICU: intensive care unit, GCS: Glasgow Coma Scale; ED: Emergency Department; LOS: length of stay; EVD: External ventricular device; MV: mechanical ventilation.

### 3.2 Second level radiographic data results

**Fig. 3:**
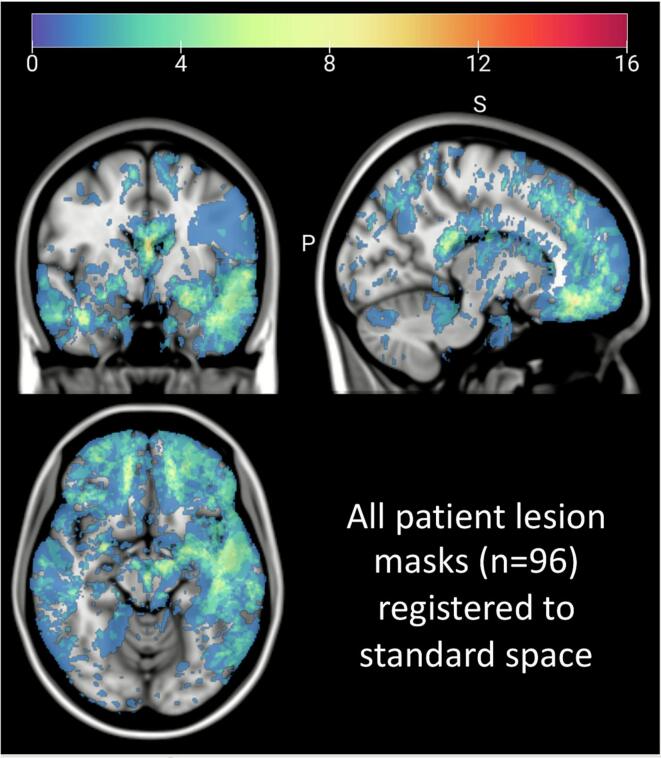
**Spatial distribution of hemorrhagic lesions included in the sample.** Simple overlay of all 96 patients’ normalized hemorrhagic lesion masks, mapped onto MNI template space. Color represents the number of patients with lesions in each voxel, with warmer colors indicating that the voxel was more commonly lesioned in the sample.

Normalized lesion mask overlays for all patients are visually displayed in Fig. 3, demonstrating the distribution and overlap of hemorrhagic lesions in our sample. Supplementary figures illustrate the distribution of patient-level gray matter parcel damage and white matter tract disconnection severity with box plots for each atlas-based parcel and tract, respectively (Supplementary Figs. 2–4).

**Fig. 4:**
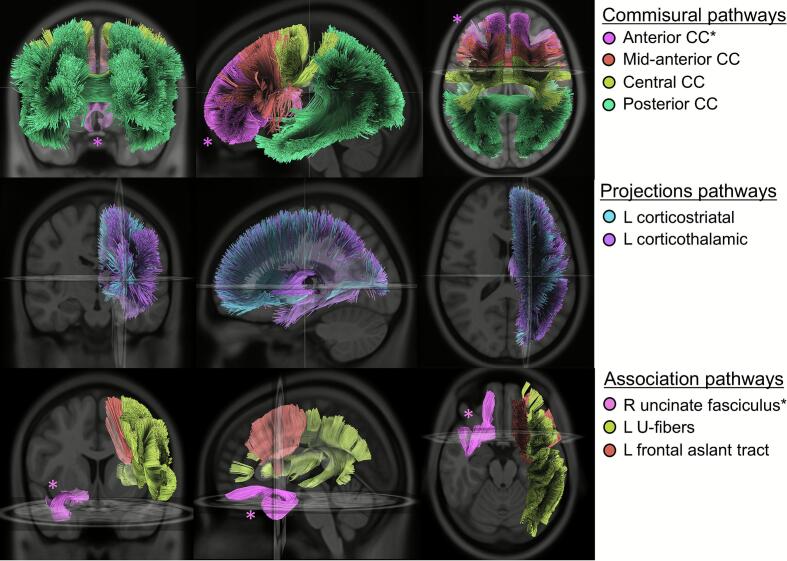
**White matter tracts associated with PSH.** Highlighted white matter tracts demonstrated significantly greater disconnection severity in patients with paroxysmal sympathetic hyperactivity (PSH) diagnosis, as detailed in Table 2. Asterisks indicate tracts for which disconnection severity was also positively correlated with a higher diagnostic likelihood score.

Table 3 lists white matter tracts with higher disconnection severity associated with PSH diagnosis. Listed tracts met Bonferroni-corrected statistical significance, with uncorrected p-values reported. The strongest effect was demonstrated in the right uncinate fasciculus, followed by four segments of the corpus callosum and multiple left hemispheric association and projection pathways. The right uncinate fasciculus and the anterior corpus callosum also correlated linearly with higher diagnosis likelihood of PSH. These tracts are visualized 3-dimensionally in Fig. 4.

**Table 3:** White matter tract disconnections associated with PSH diagnosis.

**White matter tract name**	**White matter tract category**	**Avg mean difference of disconnection severity** **PSH vs Control**	**P-value**	**Pearson coefficient** **DLT scores**	**P-value**
Right Uncinate Fasciculus (UF)	Association	20.56	0.0002*	0.413	<0.0001*
Anterior corpus callosum	Commissural	16.71	0.0005*	0.373	0.0002*
Mid-anterior corpus callosum	Commissural	13.66	0.0006*	0.313	0.0019
Posterior corpus callosum	Commissural	12.55	0.0005*	0.257	0.0116
Central corpus callosum	Commissural	12.41	0.0002*	0.270	0.0077
Left frontal aslant tract (FAT)	Association	8.52	0.0002*	0.262	0.0098
Left corticostriatal pathway (CS)	Projection	6.48	<0.0001*	0.291	0.0041
Left corticothalamic pathway (CT)	Projection	5.43	<0.0001*	0.256	0.0118
Left u-fiber pathway (U)	Association	1.16	0.0001*	0.0125	0.9036

PSH: paroxysmal sympathetic hyperactivity; DLT: diagnosis likelihood tool. *Survived Bonferroni correction for multiple comparisons.

**Fig. 5:**
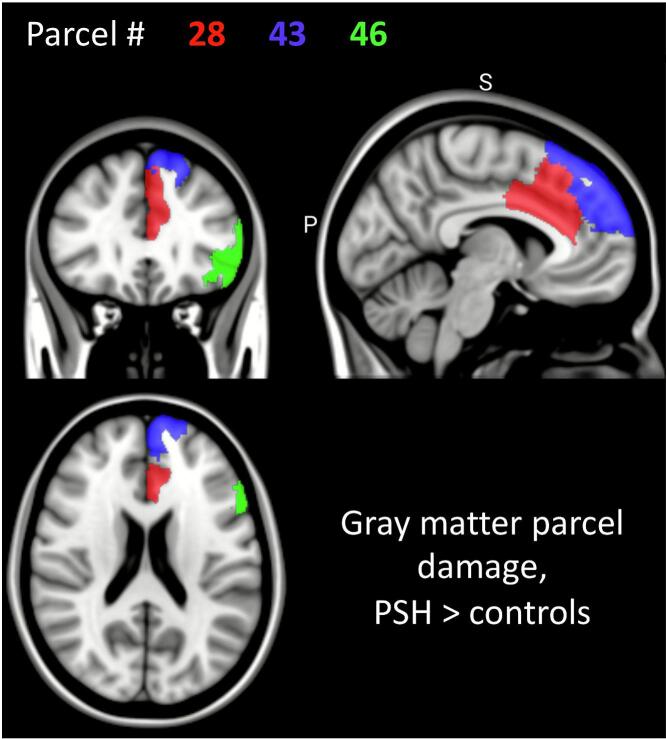
**Gray matter parcels associated with PSH.** Highlighted gray matter parcels demonstrated significantly greater damage in patients with paroxysmal sympathetic hyperactivity (PSH) diagnosis, as detailed in Table 3.

Table 4 lists gray matter parcels with significantly higher percent damage associated with PSH. Listed parcels met Bonferroni-corrected statistical significance, with uncorrected p-values reported. These include left cortical components of the default mode network and the salience/ventral attention network. These parcels are visualized in Fig. 5. In contrast to white matter tract disconnection severity, no gray matter parcels demonstrated percent damage correlations with DLT scores.

**Table 4:** Gray matter parcel damage associated with PSH diagnosis.

Gray matter **parcel**	**Avg mean difference of parcel damage PSH vs Control**	**P-value**	**Pearson coefficient for** **DLT scores**	**P-value**
43: Left PFC (DMN)	1.964	0.00005*	0.173	0.0859
46: Left PFC (DMN)	1.459	0.00029*	0.171	0.0960
28: Left ACC (Salience / ventral attention network)	0.004	0.00051*	0.041	0.691

PSH: paroxysmal sympathetic hyperactivity; DLT: diagnosis likelihood tool; PFC: prefrontal cortex; DMN: default mode network; ACC: anterior cingulate cortex. *Survived Bonferroni correction for multiple comparisons.

## 4 Discussion

### 4.1 Primary study findings

In this study, we identified SWI-based white matter tracts and gray matter parcel pathology associated with PSH in a cohort of critically ill TBI patients using connectome-based analyses of clinically acquired MRIs. Nine white matter tracts demonstrated greater disconnection severity in PSH patients, including the right uncinate fasciculus, the anterior, mid-anterior, central and posterior segments of the corpus callosum, and the left corticostriatal, corticothalamic, frontal aslant, and u-fiber pathways. Notably, disconnection severity of the right uncinate fasciculus and the anterior corpus callosum also significantly correlated with higher DLT scores, suggesting a dose–response relationship between the extent of disconnection and the likelihood of exhibiting a classic PSH phenotype. Gray matter findings were less robust, but three parcels – each located within the left prefrontal cortex and belonging to the default mode or ventral salience networks – showed significantly greater damage in PSH patients.

### 4.2 Study findings in relation to prior work

Our findings corroborate and extend prior imaging studies of post-TBI PSH using an indirect disconnection-symptom mapping approach in a comparatively large cohort. Earlier work from our group identified SWI lesions within the medial temporal lobes, prefrontal cortex and corpus callosum as associated with higher PSH likelihood (Podell et al., 2024). The present study builds on those observations by quantitatively assessing tract-specific disconnection and parcel-specific damage patterns. Consistent with our results, prior MRI and CT studies have implicated the corpus callosum, cingulate cortex and subcortical white matter in PSH after TBI (Hinson et al., 2015, Lv et al., 2010). However, our study offers several methodological advantages. Our analysis represents a novel application of connectome-based lesion mapping, leveraging standard clinical MRI sequences to infer network‑level structural disruptions associated with an important but under-studied neurologic complication of TBI. As a result, we were able to draw from a large single institution repository of observational clinical data to perform an indirect disconnection-symptom mapping study.

The high prevalence of PSH in our sample likely reflects several intentional design features. First, defining PSH based on sustained use of first-line pharmacologic pharmacologic treatments in the acute setting likely results in a more inclusive PSH population compared to studies set in subacute and chronic phases of injury. Second, our selection criteria – requiring ICU admission for at least three days and hospital admission for at least two weeks – enriched for PSH by selecting patients with more prolonged critical illness and more severe brain injuries. This pattern is consistent with our previous report of a 37 % prevalence (Podell et al., 2021), which exceeds the 8–10 % reported in broader acute brain injury cohorts (Baguley et al., 2014). Notably, lower prevalence estimates may consider other causes of acute brain injury such as stroke, hypoxic-ischemic injury, or encephalitis (which carry lower risk for PSH compared to TBI) and more chronic phases of care. Additionally, requiring that patients undergo MRI as part of clinical care further biased the cohort toward those with more severe or clinically perplexing injuries warranting evaluation for diffuse axonal injury or occult ischemia. Together, these factors explain the 50 % PSH rate observed in this study.

### 4.3 Pathophysiologic implications

Our results provide new insights into the neurobiological mechanisms underlying PSH after TBI. Disconnection of the uncinate fasciculus was strongly associated with PSH clinical diagnosis and DLT scores. As a major limbic-frontal pathway and a component of the central autonomic network, (Von Der Heide et al., 2013, Reisert, 2021) the uncinate fasciculus supports top-down regulation of the amygdala through reciprocal connections within prefrontal regions involved in emothional salience and sensory processing (Avecillas-Chasin et al., 2023, Johnson et al., 2011, Von Der Heide et al., 2013). It integrates emotional salience, assigns value to stimuli, and mediates associative memory within the lateral amygdala. Dysfunction of this tract has been linked to impaired fear modulation and heightened amygdala responsiveness in disorders such as PTSD (Koch et al., 2017). Given its downstream projections to the centromedial amygdala—an autonomic control hub (Chokroverty & Bhatia, 2022)—its disruption may reduce prefrontal inhibition of limbic autonomic circuitry, resulting in exaggerated sympathetic responses to non‑nociceptive stimuli (“allodynic hyperreactivity”) in PSH (Chokroverty and Bhatia, 2022). Its disruption may reduce prefrontal inhibition of limbic autonomic circuitry, resulting in exaggerated sympathetic responses to non-nociceptive stimuli (“allodynic hyperreactivity”) in PSH.

The lateralization of our findings offers additional mechanistic clues. Prior research suggests that parasympathetic regulation is predominantly mediated by the dominant (left) hemisphere, whereas sympathetic regulation is right hemispheric (Beissner et al., 2013, Diedrich et al., 2009, Guo et al., 2016, Harper et al., 2000, Mazzola et al., 2023, Wittling et al., 1998a, Wittling et al., 1998b). The association between PSH and right uncinate fasciculus disruption aligns with this autonomic lateralization framework, supporting a model in which right hemisphere uncinate injury produces disinhibition of limbic autonomic outputs, generating excessive sympathetic activity. Further, the right uncinate fasciculus has been more strongly implicated in emotional and social processing, while the left is more involved in language and semantic processing (Harvey et al., 2013, Toller et al., 2022). Studies of PTSD report right-sided uncinate abnormalities linked to greater anxiety and symptom severity (Koch et al., 2017, O’Doherty et al., 2018). By contrast, left-sided cortical pathology implicated in this study may unbalance autonomic activity by reducing parasympathetic output. Supporting this idea, left hemispheric strokes have been associated with heart rate variability metrics of reduced parasympathetic activation (Constantinescu et al., 2019, Meyer et al., 2004) and left cingulate cortex degeneration in behavioral variant frontotemporal dementia has been associated with diminished cardiac vagal tone (Guo et al., 2016). Taken together, these observations suggest that PSH may reflect a dual process: sympathetic hyperactivity as a release phenomenon due to right-sided white matter tract damage, and parasympathetic hypoactivity resulting from left-sided cortical injury. However, laterality effects in our study are not definitive; using a more liberal threshold (uncorrected α = 0.05), disconnection severity of the *left* uncinate fasciculus is also associated with PSH and correlates with DLT scores, possibly reflecting individual differences in brain structure–function relationships.

PSH patients in our cohort were younger, had lower GCS scores, and more frequently sustained MVCs, rather than falls. High-velocity injuries, common in PSH patients, are associated with increased shear stress and diffuse axonal injury – a known risk factor for PSH (Podell et al., 2024, 2021). Consistent with the excitatory-inhibitory ratio model, diffuse axonal injury likely contributes to PSH by disrupting white matter tracts. However, because younger age, diffuse axonal injury, lower GCS, and MVCs are interrelated and all associated with PSH, causality cannot be assumed. Developmental factors also may contribute. The uncinate fasciculus is among the last major white matter tracts to mature, with development extending into the third decade of life (Hasan et al., 2009, Lebel et al., 2008, Olson et al., 2015). Because PSH patients in our cohort were significantly younger than controls, incomplete tract maturation may increase vulnerability to injury-induced disconnection, potentially predisposing younger individuals to PSH. Age- and GCS-adjusted logistic regression analyses are presented in the supplementary material (Supplementary Tables 1–3). Notably, a multivariable logistic regression analysis identified disconnection severity of the posterior corpus callosum and right uncinate fasciculus as independent predictors of PSH.

### 4.4 Study limitations

This study has a number of limitations. First, lesions were manually traced – a gold standard, but labor-intensive and prone to human error, limiting scalability to larger datasets. Second, severe injury-related brain deformation posed challenges for spatial normalization, which is critical for connectome-based analyses. We achieved 82 % success in image registration to the template space (Fig. 1) based on manual inspection of cortical and subcortical landmarks. Although quantitative metrics could reduce bias, manual review remains standard in populations with significant anatomic distortion, where automated similarity measurements often fail. Despite similar registration-related exclusion rates for PSH cases and controls, systematic bias may persist, particularly in regions such as the skull base or midline structures, and in cases of extreme deformation. These factors, along with image resolution constraints limit conclusions about small structures such as specific nuclei. Future improvements in registration algorithms and machine-learning-based lesion identification reduce bias and improve scalability.

Additionally, in connectome-based analyses, group-level results are constrained by the chosen atlas’s definition of tracts, parcels, and networks (Nozais et al., 2023). The chosen Schaefer atlas, used for gray matter parcellation, applies local gradient and global similarity approaches using task and resting state fMRI data to parcel cortical gray matter into distinct functional units (Schaefer et al., 2018). While this reduces dimensionality compared to a voxel-wise approach, the large number of small parcels limits cross-subject comparisons. Future work may aggregate parcels into network-level measures of lesion burden.

Limitations related to the use of the HCP-842 white matter tract atlas include its inability to capture individual variability in white matter anatomy (a challenge inherent to indirect disconnection-symptom mapping) and its reliance on expert judgment for tract delineation. Further, this atlas considers the corpus callosum as a single structure, failing to capture its functional and anatomic anterior to posterior distinctions (Yeh et al., 2018). While the Lesion Quantification Toolkit addresses this limitation by further subdividing the corpus callosum into five anterior to posterior divisions (Fischl et al., 2002), their borders are somewhat arbitrary and fail to capture individual differences in structure and function. Several subdivisions of the corpus callosum appear relevant to PSH in our study, warranting targeted investigation of the role of corpus callosum disconnection in PSH pathophysiology.

PSH occurs not only in TBI but also in other forms of brain injury, commonly in hypoxic-ischemic injury, encephalitis and stroke, and less commonly as a result of brain tumors and other focal lesions (Meyfroidt et al., 2017). Our analysis focused on post-TBI PSH and modeled network effects of hemorrhagic lesions detected by SWI, chosen for its sensitivity to diffuse axonal injury – a strong predictor of PSH – and prior evidence linking SWI lesions to PSH (Podell et al., 2024). It is important to consider the neuropathologic correlations of SWI lesions when interpreting this study, as lesion-symptom mapping studies show that lesion etiology (i.e. stroke versus tumor) influences functional outcome (van Grinsven et al., 2023). SWI detects blood product (hemosiderin) deposition in the brain. In the context of TBI, SWI lesions reflect either macrohemorrhages (contusions) or microhemorrhages from diffuse axonal injury. Although SWI is more sensitive than other sequences (e.g. FLAIR, DWI) for detecting traumatic axonal injuries, it may underestimate non-hemorrhagic axonal injury (Benson et al., 2012, Geurts et al., 2012, Sharp and Ham, 2011, Spitz et al., 2013). Histopathologic studies confirm that SWI lesions correspond to axonal injury but do not indicate complete destruction of affected regions (Benson et al., 2012). Diffusion tensor imaging offers complementary measures of microstructural integrity and can detect pathology in normal-appearing white matter (Andreasen et al., 2020). SWI lesion visibility also depends on magnet strength and timing post-injury (Környei et al., 2021, Luccichenti et al., 2010), neither of which were here. Future prospective, multimodal imaging studies are needed to confirm and expand upon these findings.

While our findings offer insight into post-TBI PSH pathophysiology, we would caution against extrapolating these results to other clinical scenarios. In conditions such as hypoxic-ischemic injury or encephalitis, cytotoxic injury visible on different MRI sequences and in distinct brain regions may contribute more strongly to PSH pathophysiology than hemorrhagic lesions. Even within TBI, traumatic injuries such as contusions and shear injury exhibit heterogeneous imaging features, and by limiting our analysis to SWI lesions, we may fail to capture contributions from edema or ischemia extending beyond hemorrhagic areas. Future studies incorporating multiple MRI sequences before connectome integration could yield results more applicable to other etiologies where FLAIR or diffusion abnormalities predominate.

Additionally, this study is descriptive, rather than predictive and reflects a single-center retrospective cohort, which is prone to selection bias. Larger, multicenter studies are needed to develop imaging-based predictive models that identify high risk patients for early intervention. The diversity of brain injury mechanisms and anatomical patterns producing PSH underscores the need to define a final common pathway for this syndrome. While our work highlights TBI-associated structural injury patterns that predispose to PSH, functional neuroimaging studies are needed to truly understand its dynamic neural network correlates.

Despite improved diagnostic standardization with the PSH-AM (Baguley et al., 2014), PSH remains a subjective clinical diagnosis vulnerable to bias and human error. For example, clinicians may diagnose PSH more frequently in patients with diffuse axonal injury, reinforcing observed associations. Although the PSH-AM DLT score offers a more granular measure of diagnostic likelihood, it remains subjective and challenging to apply retrospectively. Objective, quantifiable diagnostic and monitoring tools are needed to link brain injury patterns with their physiologic autonomic expressions. Future research should explore correlations between imaging findings and with data-driven physiologic metrics of autonomic imbalance, which may more precisely define the contributions of various pathways to PSH pathophysiology. Quantitative physiologic signatures of PSH may include heart rate variability and artificial intelligence-assisted physiologic data modeling (Baguley et al., 2006, Podell et al., 2022).

## 5 Conclusions

In this study, we analyzed a large single-center cohort of critically ill TBI patients using indirect disconnection-symptom mapping to investigate PSH. We identified a novel association between PSH and disconnection of the right uncinate fasciculus – a tract linking the frontal lobe and amygdala – suggesting a mechanism for sympathetic overactivity via disinhibition of subcortical autonomic nodes. The corpus callosum also demonstrated strong associations with PSH, consistent with prior work. Additionally, left-sided frontal cortical and white matter tract damage correlated with PSH, implicating disruption of supratentorial parasympathetic control. These preliminary findings highlight network-level injury patterns that may predispose to PSH and warrant further investigation through multimodal and functional imaging approaches.

## CRediT authorship contribution statement

**Eric W Moffet:** Writing – review & editing, Writing – original draft, Visualization, Project administration, Methodology, Investigation, Formal analysis. **Sancharee Hom Chowdhury:** Writing – review & editing, Visualization, Methodology, Investigation, Formal analysis. **Ediel Almeida:** Writing – review & editing, Methodology, Investigation, Formal analysis, Data curation. **Xiangxiang Kong:** Writing – review & editing, Methodology, Investigation, Formal analysis. **Lujie Chen:** Writing – review & editing, Visualization, Supervision, Methodology, Investigation, Funding acquisition, Formal analysis. **Jiachen Zhuo:** Writing – review & editing, Validation, Supervision, Methodology. **Nicholas A Morris:** Writing – review & editing, Supervision, Resources, Methodology, Conceptualization. **Gunjan Y Parikh:** Writing – review & editing, Supervision, Resources, Data curation, Conceptualization. **Neeraj Badjatia:** Writing – review & editing, Supervision, Resources, Methodology, Funding acquisition, Conceptualization. **Jamie E Podell:** Writing – review & editing, Writing – original draft, Visualization, Validation, Supervision, Software, Resources, Project administration, Methodology, Investigation, Funding acquisition, Formal analysis, Data curation, Conceptualization.

## Declaration of competing interest

The authors declare that they have no known competing financial interests or personal relationships that could have appeared to influence the work reported in this paper.

## Data Availability

Data will be made available on request.
