# Supplementary material for: Fronto-limbic disconnection correlates with paroxysmal sympathetic hyperactivity following traumatic brain injury: An indirect disconnection-symptom mapping study
Source: Neuroimage Clin. 2025 Dec 23;49:103937. doi: 10.1016/j.nicl.2025.103937 (PMC12811682; doi:10.1016/j.nicl.2025.103937)

**Supplementary Figure 1:** Registration quality control examples

**Supplementary Table 1:** Impact of white matter tract disconnection severity on PSH diagnosis, adjusted for age and ICU admission GCS

| **White matter tract** | **Adjusted Odds Ratio** | **95% CI, low** | **95% CI, high** | **P-value** |
| --- | --- | --- | --- | --- |
| Right Uncinate Fasciculus | 1.026 | 1.000 | 1.052 | 0.048* |
| Anterior corpus callosum | 1.013 | 0.986 | 1.039 | 0.350 |
| Mid-anterior corpus callosum | 1.021 | 0.995 | 1.048 | 0.121 |
| Posterior corpus callosum | 1.047 | 1.005 | 1.091 | 0.030* |
| Central corpus callosum | 1.028 | 0.997 | 1.061 | 0.081 |
| Left frontal aslant tract | 1.036 | 0.994 | 1.080 | 0.094 |
| Left corticostriatal tract | 1.022 | 0.991 | 1.053 | 0.170 |
| Left corticothalamic tract | 1.096 | 1.009 | 1.190 | 0.030* |
| Left u-fiber pathway | 1.0410 | 0.964 | 1.124 | 0.305 |

For each white matter tract identified in Table 2, we fit logistic regression models adjusting for Age and ICU admission GCS, to predict a clinical diagnosis of PSH. Right uncincate fasciculus, posterior corpus callosum, and left corticothalamic tract retained statistical significance after this adjustment, albeit with a more liberal unadjusted threshold.

**Supplementary Table 2:** Independent predictors of PSH diagnosis

| **Predictor** | **Odds Ratio** | **95% CI, low** | **95% CI, high** | **P-value** |
| --- | --- | --- | --- | --- |
| Age | 0.92 | 0.882 | 0.953 | <0.001 |
| Posterior corpus callosum | 1.06 | 1.01 | 1.11 | 0.025 |
| Right uncinate fasciculus | 1.02 | 0.95 | 1.05 | 0.112 |

To further supplement the above analysis, we performed a multivariable stepwise logistic regression analysis using a forward search method to select predictors from the tracts listed in Table 2 and age, sex, and GCS.

**Supplementary Table 3:** Impact of gray matter parcel damage on PSH diagnosis, adjusted for age and ICU admission GCS

| **Gray matter parcel** | **Adjusted Odds Ratio** | **95% CI, low** | **95% CI, high** | **P-value** |
| --- | --- | --- | --- | --- |
| 43: Left PFC (DMN) | 1.667 | 0.675 | 4.117 | 0.268 |
| 46: Left PFC (DMN) | 1.086 | 0.8785 | 1.342 | 0.447 |
| 28: Left ACC (Salience/ventral  attention network) | 1.005 | 0.816 | 1.237 | 0.964 |

For each gray matter parcel identified in Table 3, we fit logistic regression models adjusting for Age and ICU admission GCS, to predict a clinical diagnosis of PSH. No parcels retained statistical seignificance after this adjustment.

**
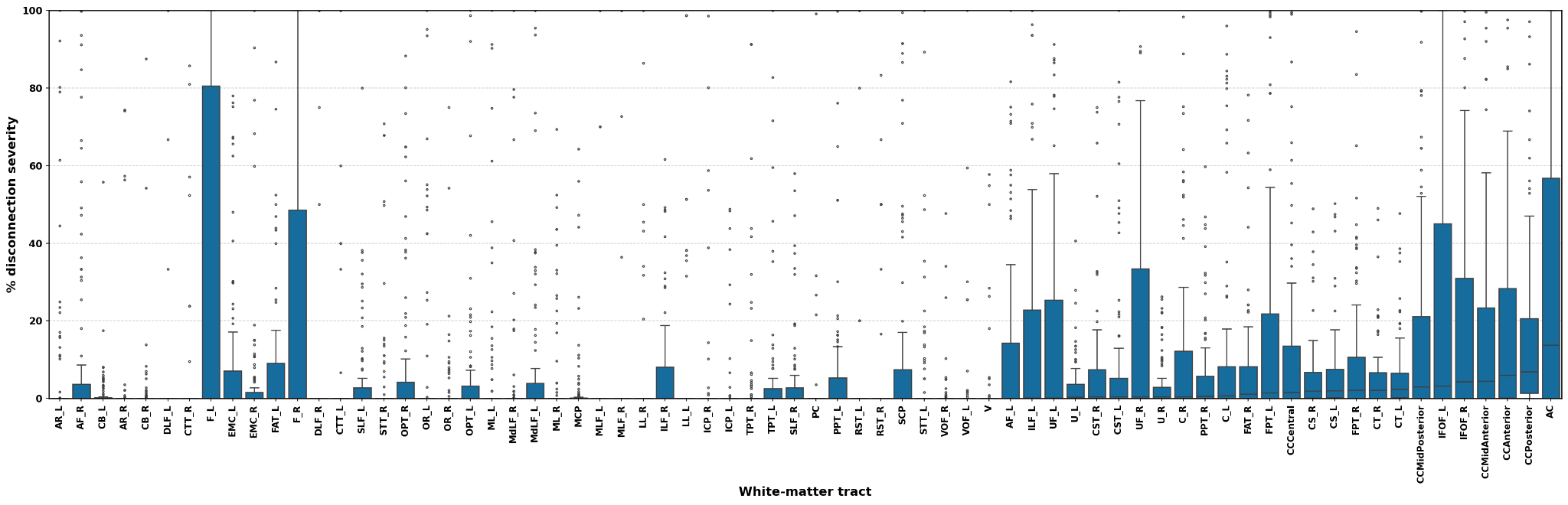
Supplementary Figure 3:** Distribution of white matter tract disconnection severity across all patients

**Supplementary Figure 4:** Distribution of cortical gray matter parcel damage across all patients


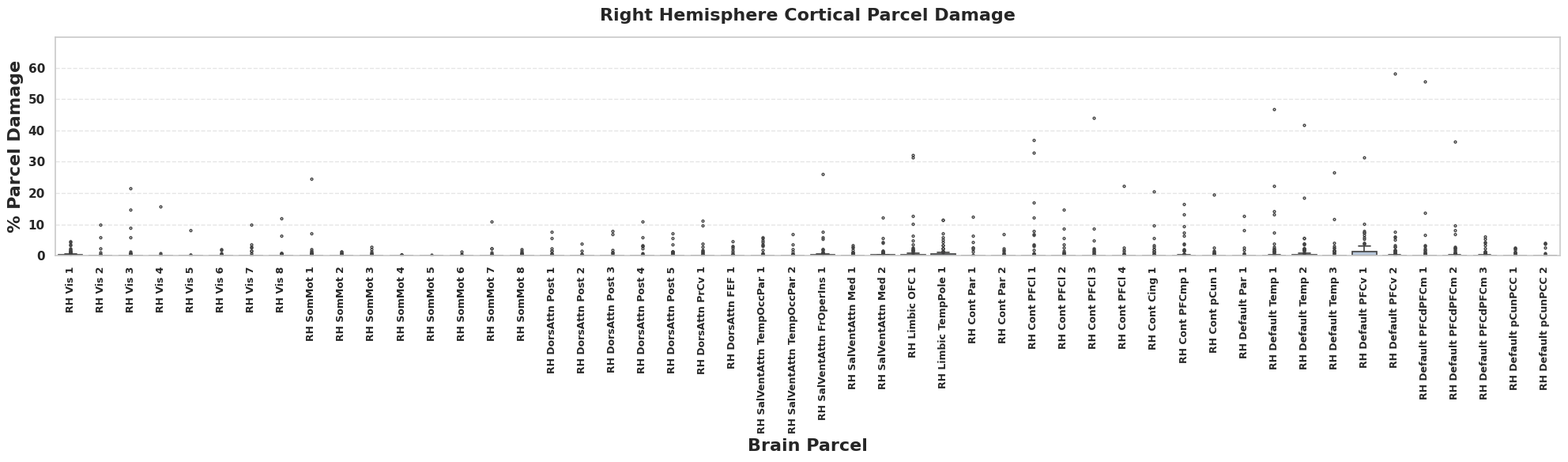

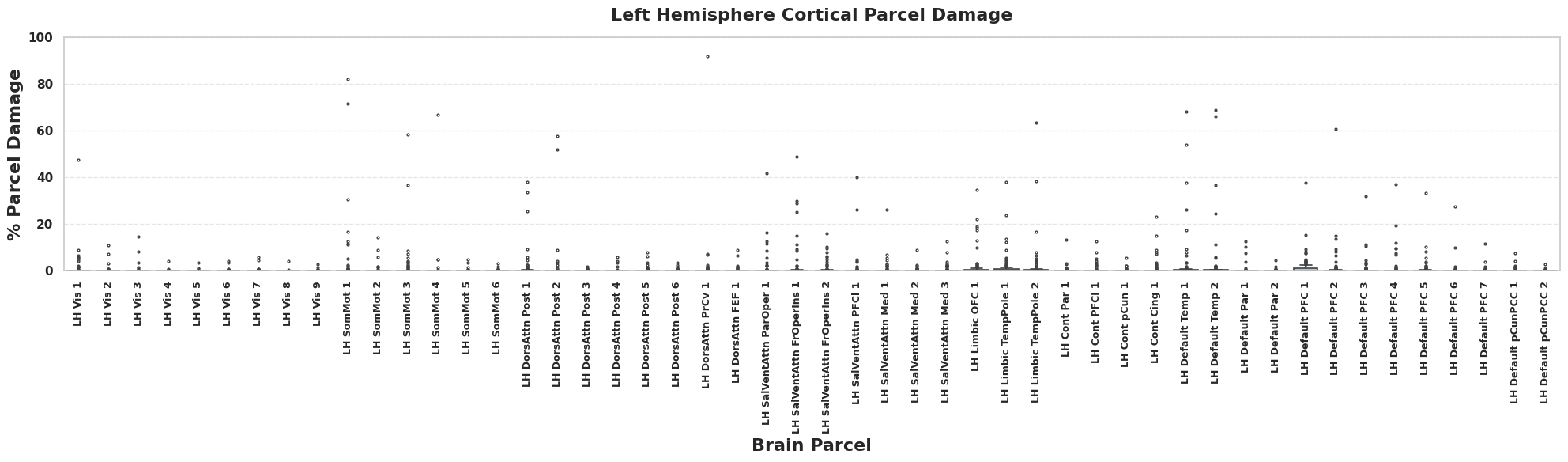


**Supplementary Figure 5:** Distribution of subcortical and cerebellar gray matter parcel damage across all patients


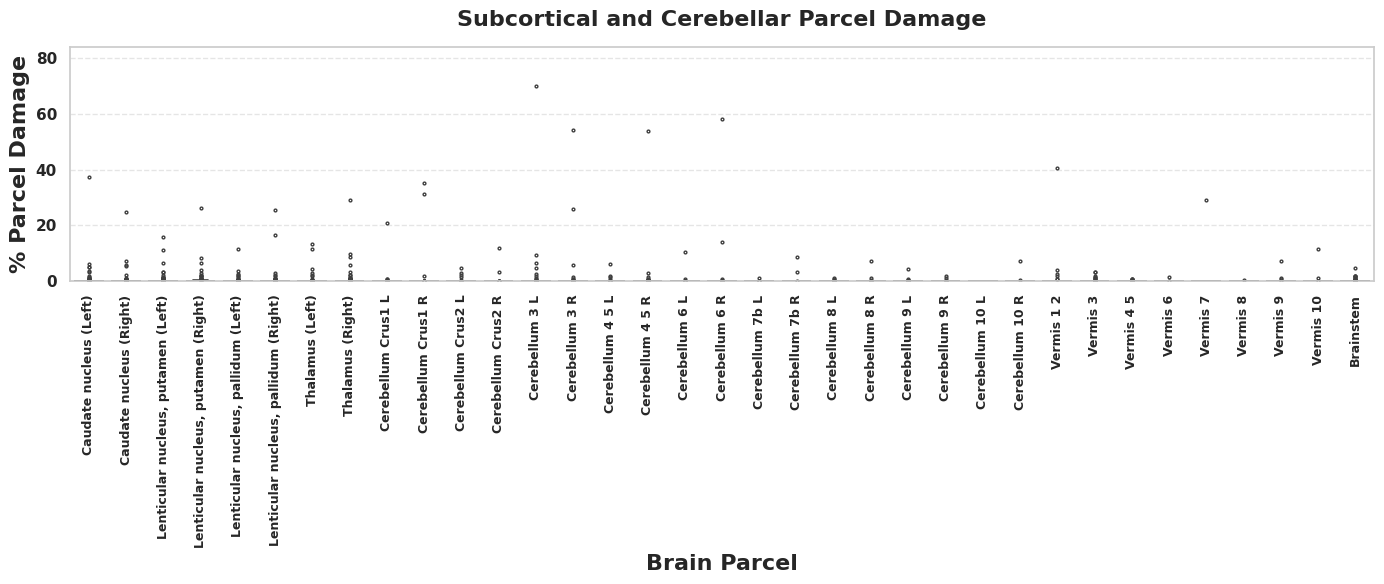

Supplement: Supplementary Data 1 [file mmc1.docx]
